# Supplementary material for: A plant tendril mimic soft actuator with phototunable bending and chiral twisting motion modes
Source: Nat Commun. 2016 Dec 22;7:13981. doi: 10.1038/ncomms13981 (PMC5192217; doi:10.1038/ncomms13981)
Supplement: Supplementary Information — Supplementary Figures, Supplementary Methods, Supplementary References [file ncomms13981-s1.pdf]

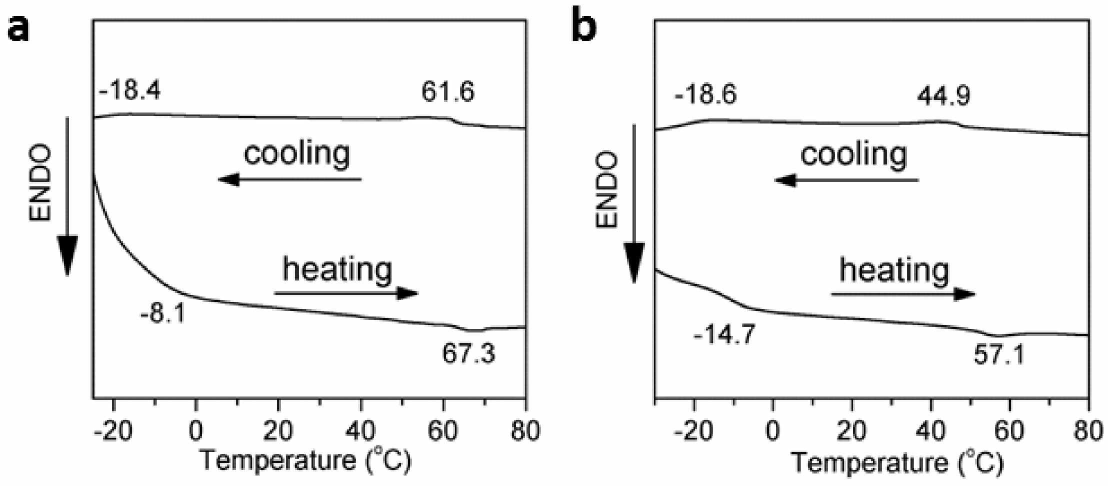

Supplementary Figure 1 | DSC curves of two LCE films. (a) PMHS-AZO46-MBB/YHD796 top layer film, (b) PMHS-MBB/YHD796 bottom layer film.

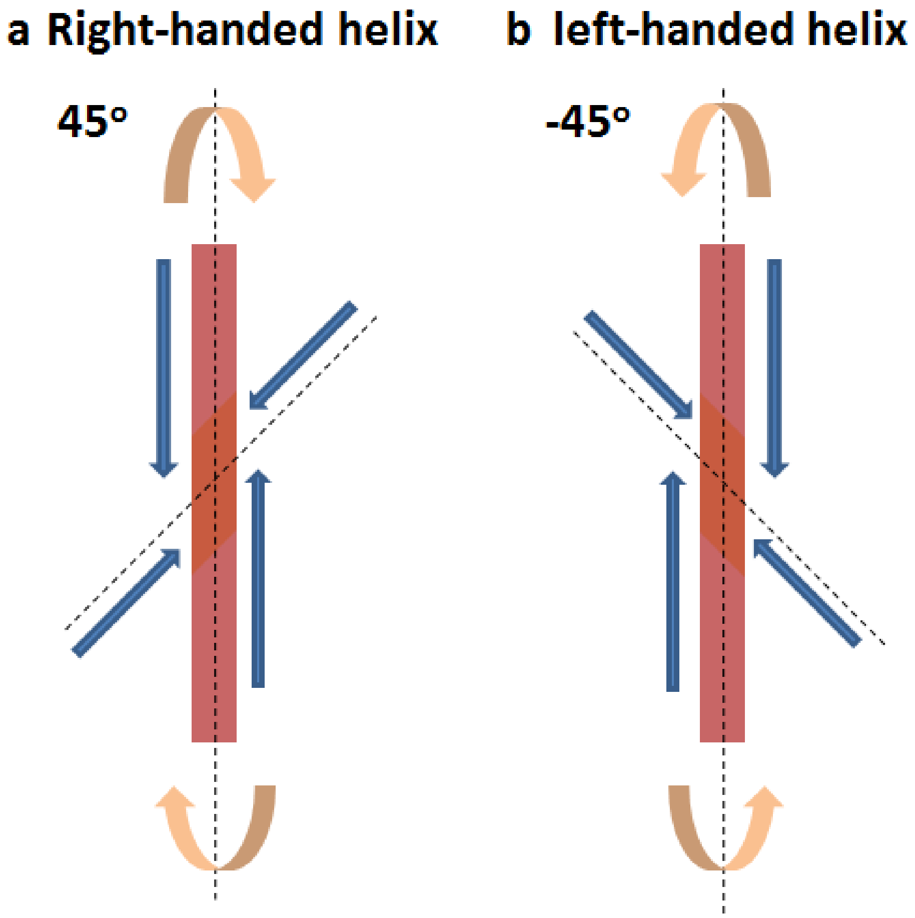

Supplementary Figure 2 | Schematic illustration of twisting deformations of the bilayer LCE ribbons. (a) The bilayer LCE ribbon with a 45° angle between the top and bottom layer, (b) The bilayer LCE ribbon with a -45° angle between the top and bottom layer.

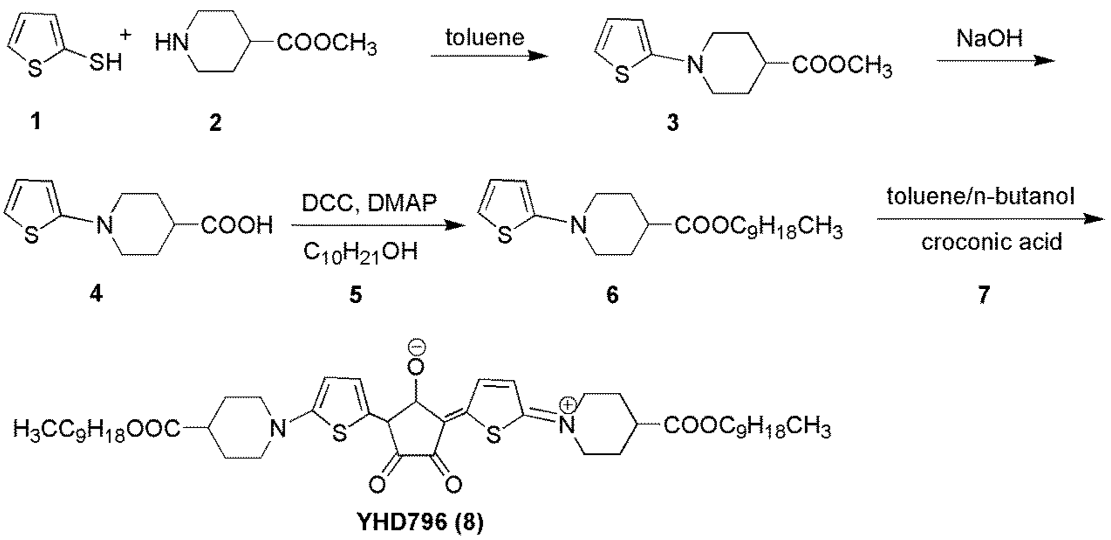

Supplementary Figure 3 | Synthetic route of monomer YHD796.

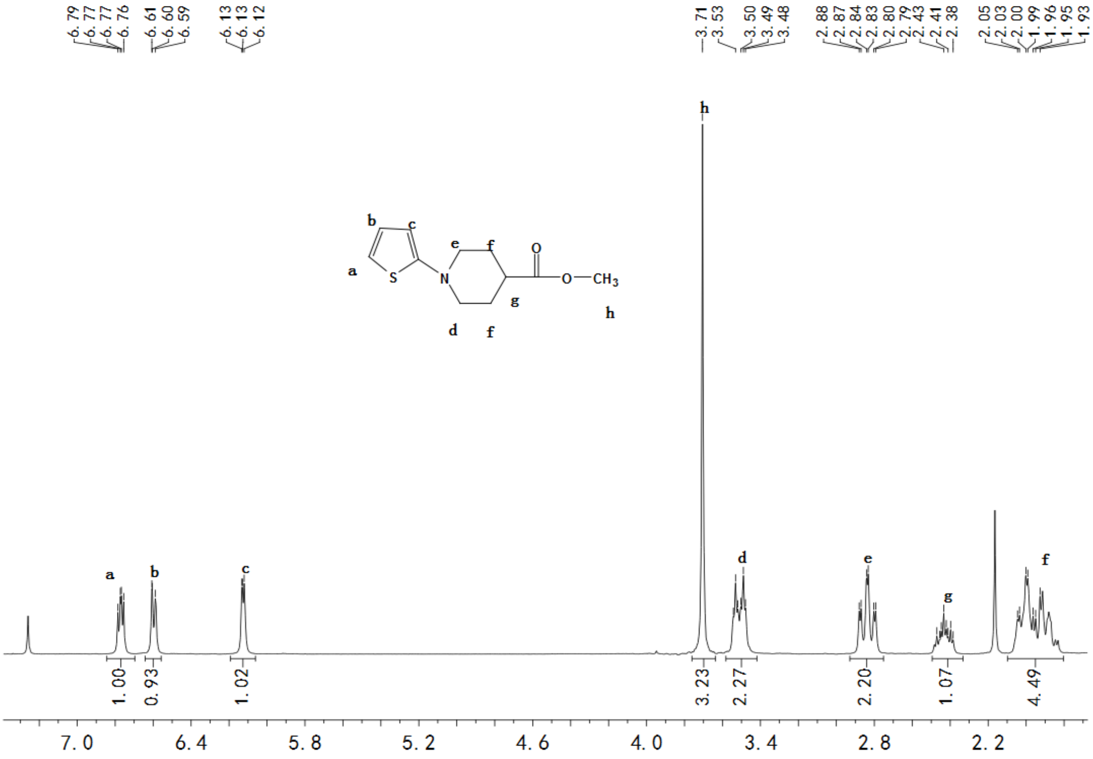

Supplementary Figure 4 | ^{1}H NMR spectrum of Compound 3.

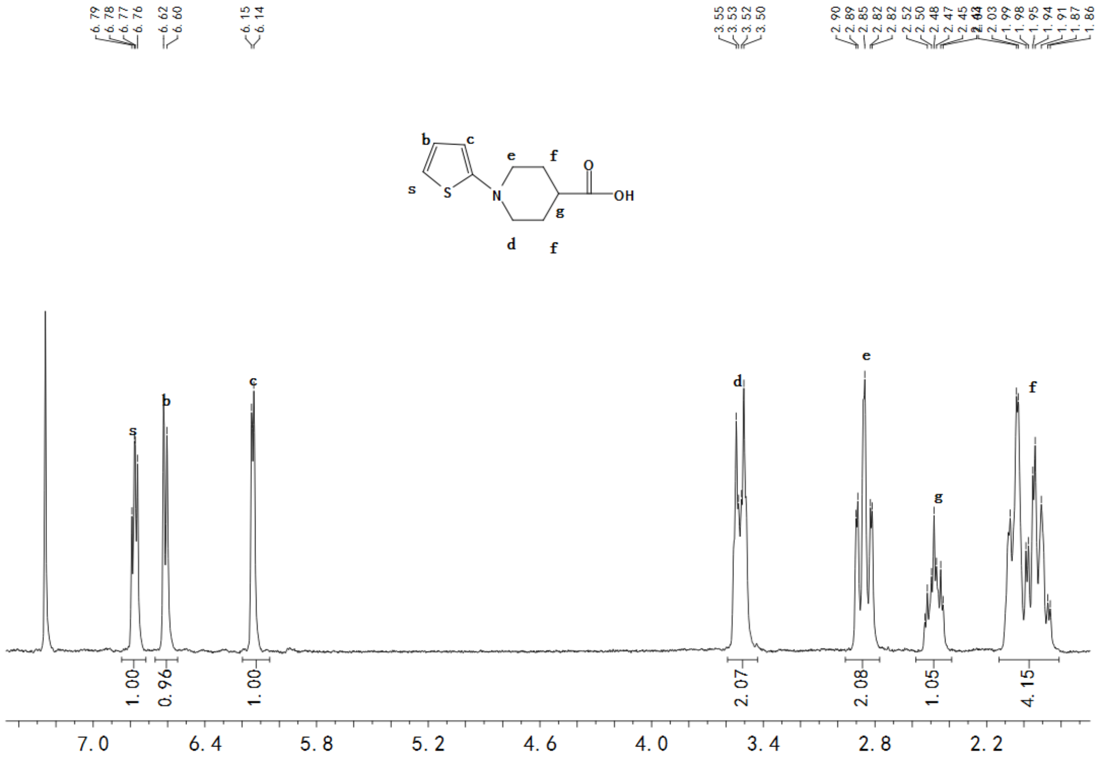

Supplementary Figure 5 | ^{1}H NMR spectrum of Compound 4.

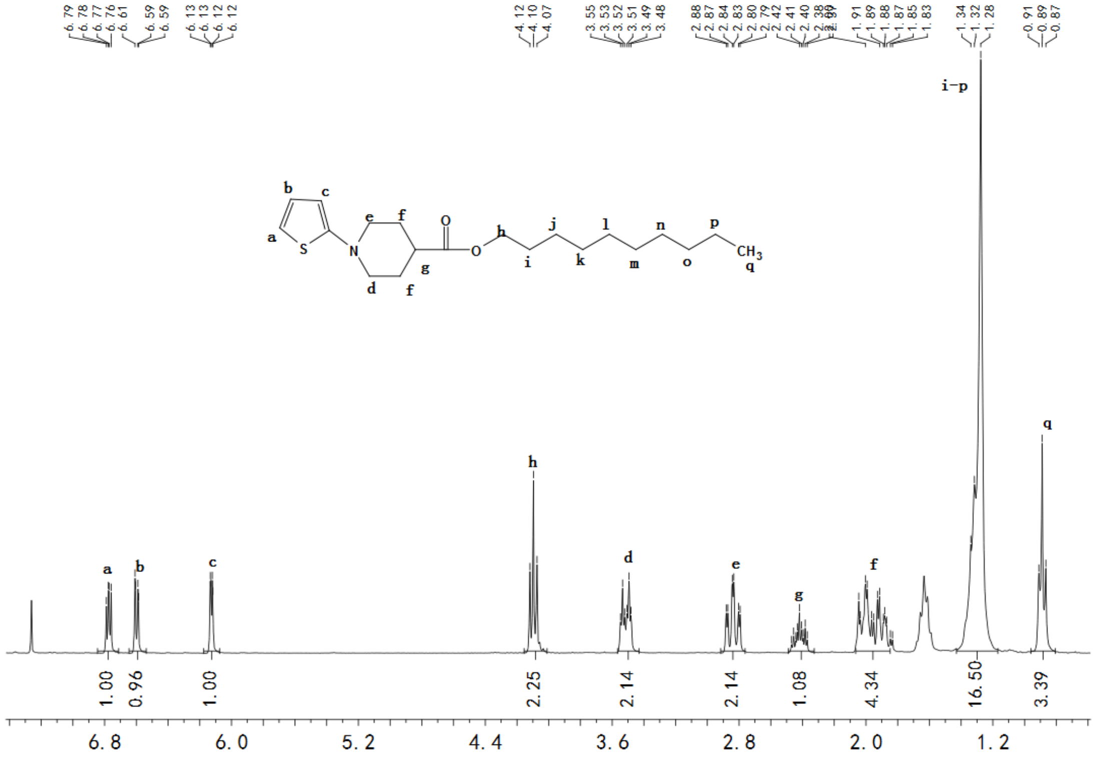

Supplementary Figure 6 | ^{1}H NMR spectrum of Compound 6.

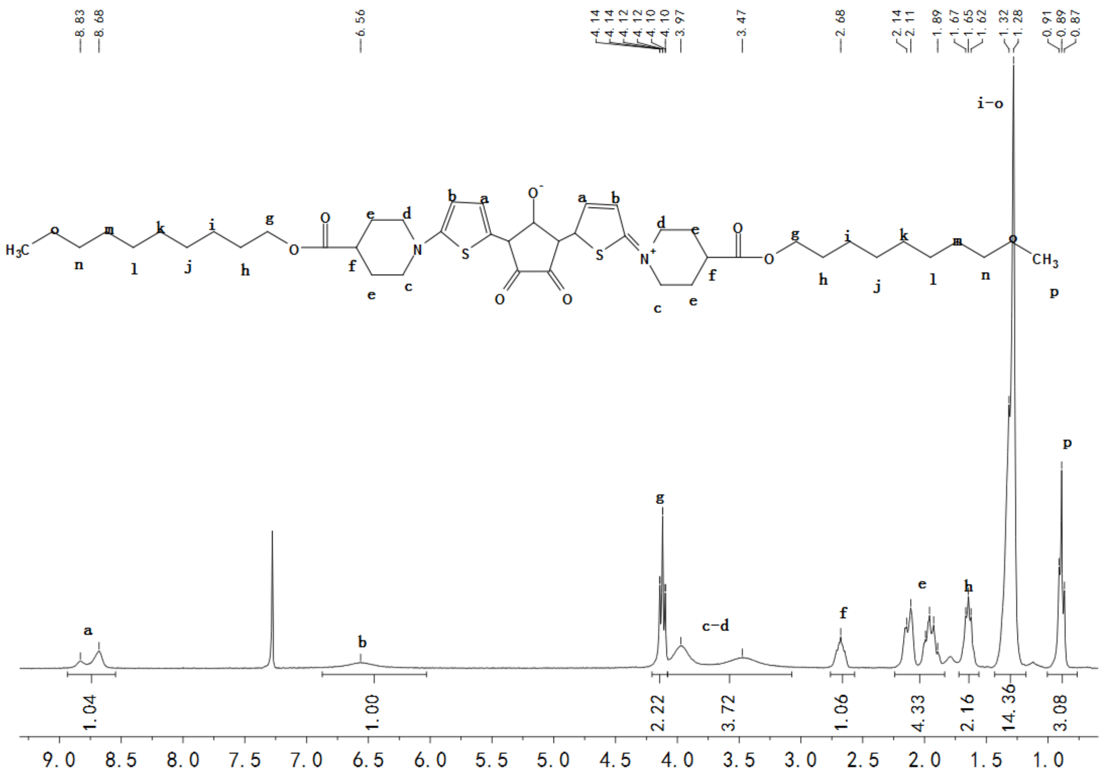

Supplementary Figure 7 | ^{1}H NMR spectrum of YHD796.

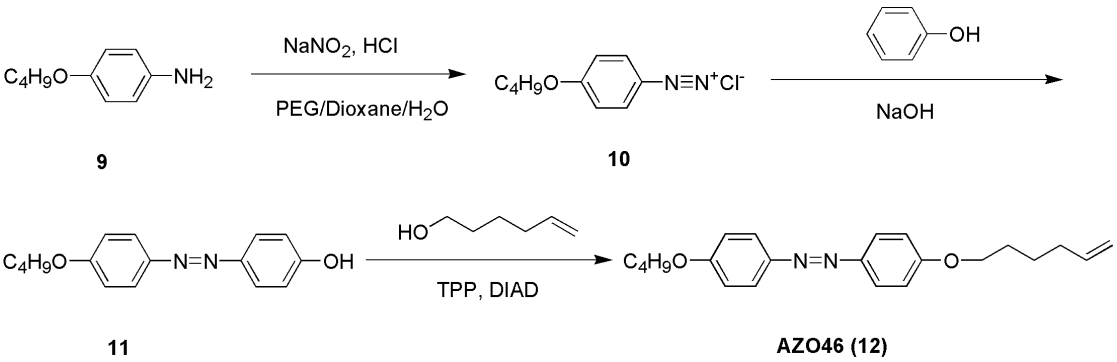

Supplementary Figure 8 | Synthetic route of monomer AZO46.

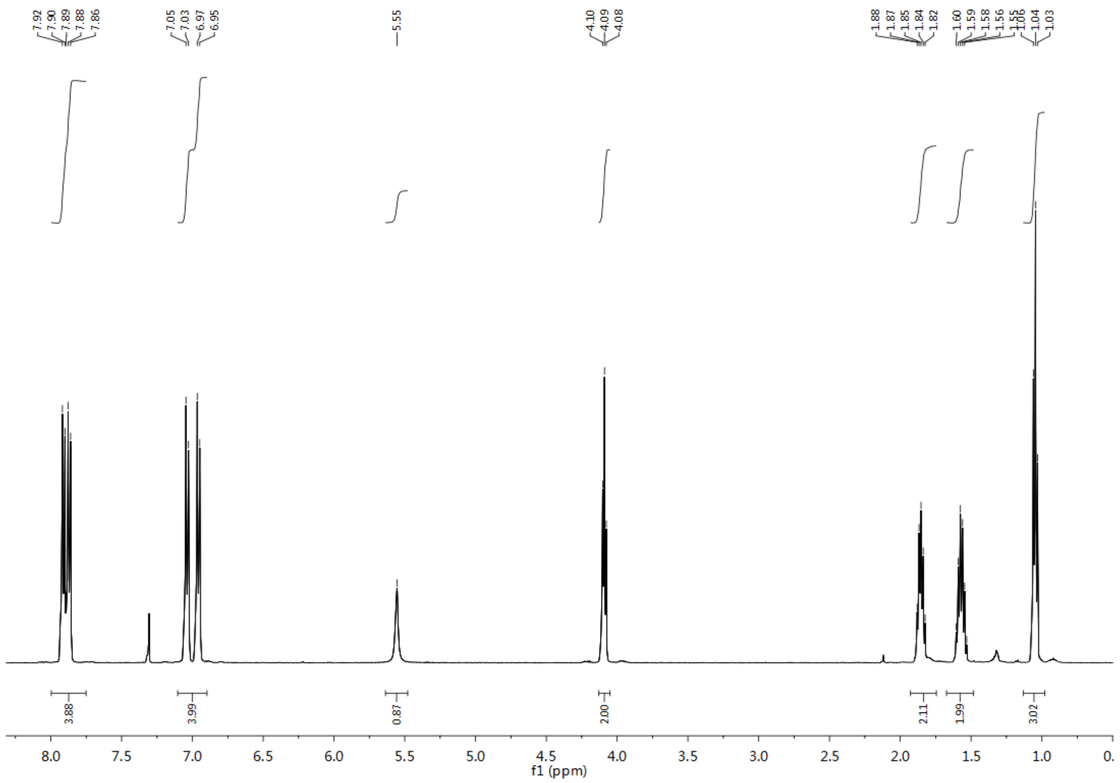

Supplementary Figure 9 | ^{1}H NMR spectrum of compound 11.

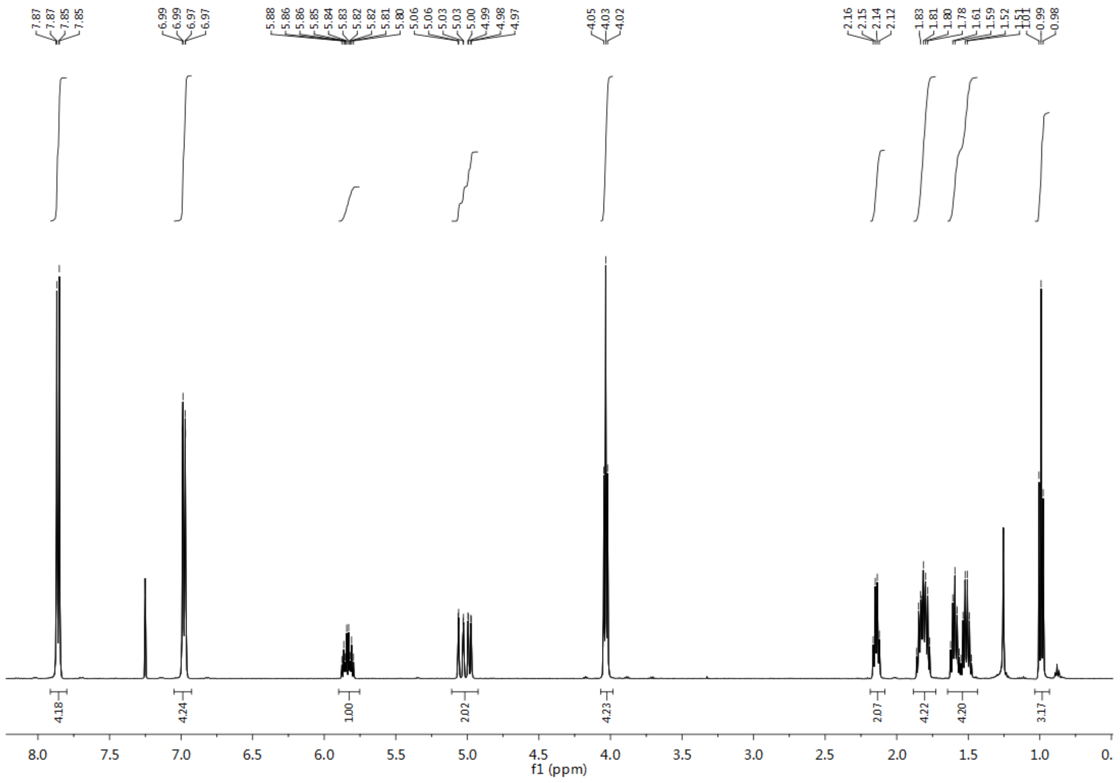

Supplementary Figure 10 | ^{1}H NMR spectrum of compound AZO46.

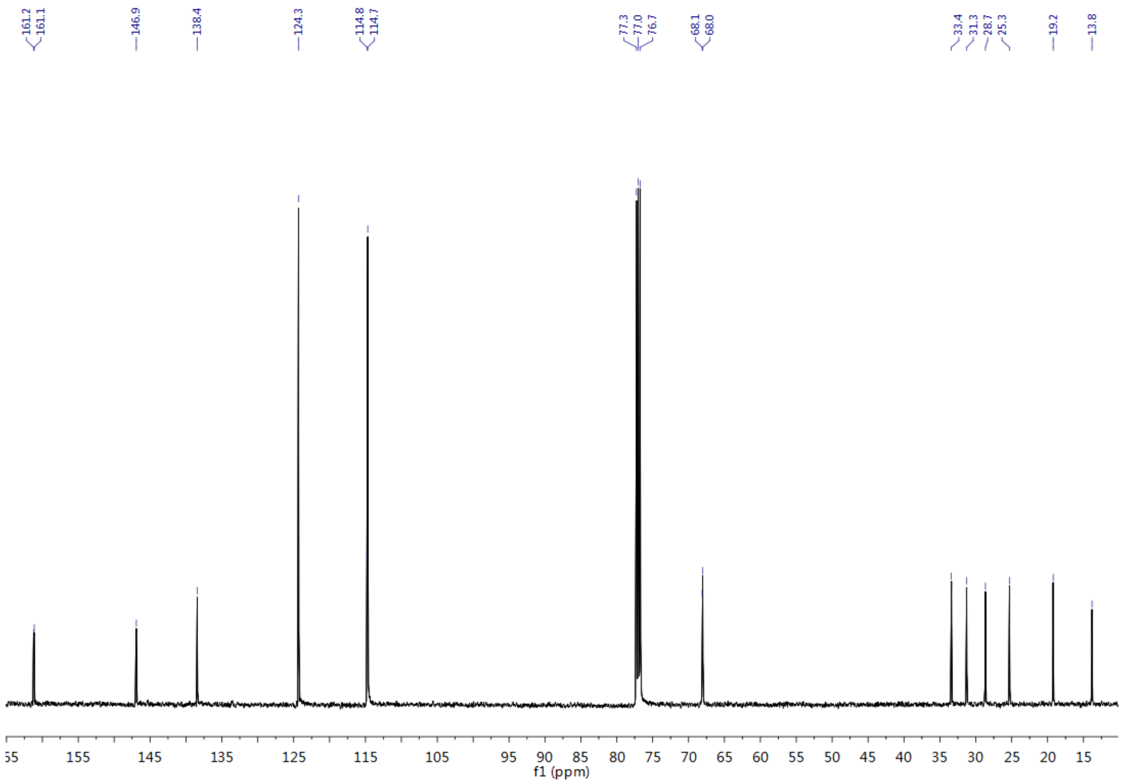

Supplementary Figure 11 | ^{13}C NMR spectrum of compound AZO46.

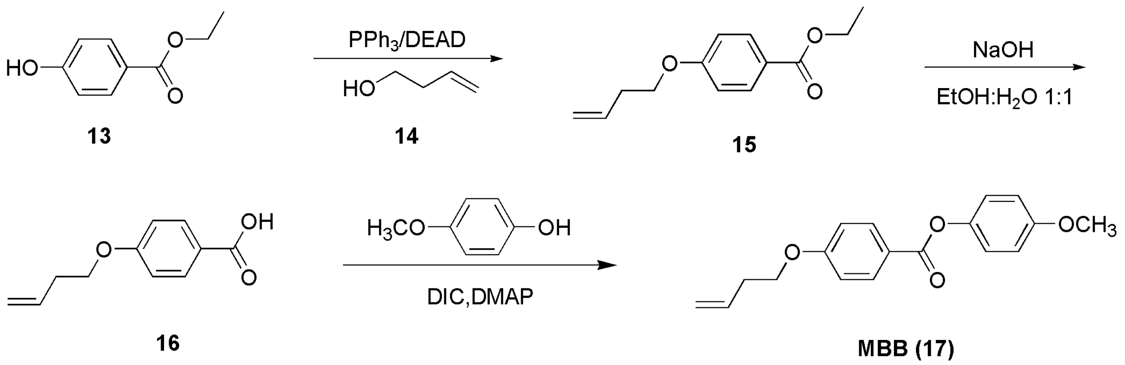

Supplementary Figure 12 | Synthetic route of monomer MBB.

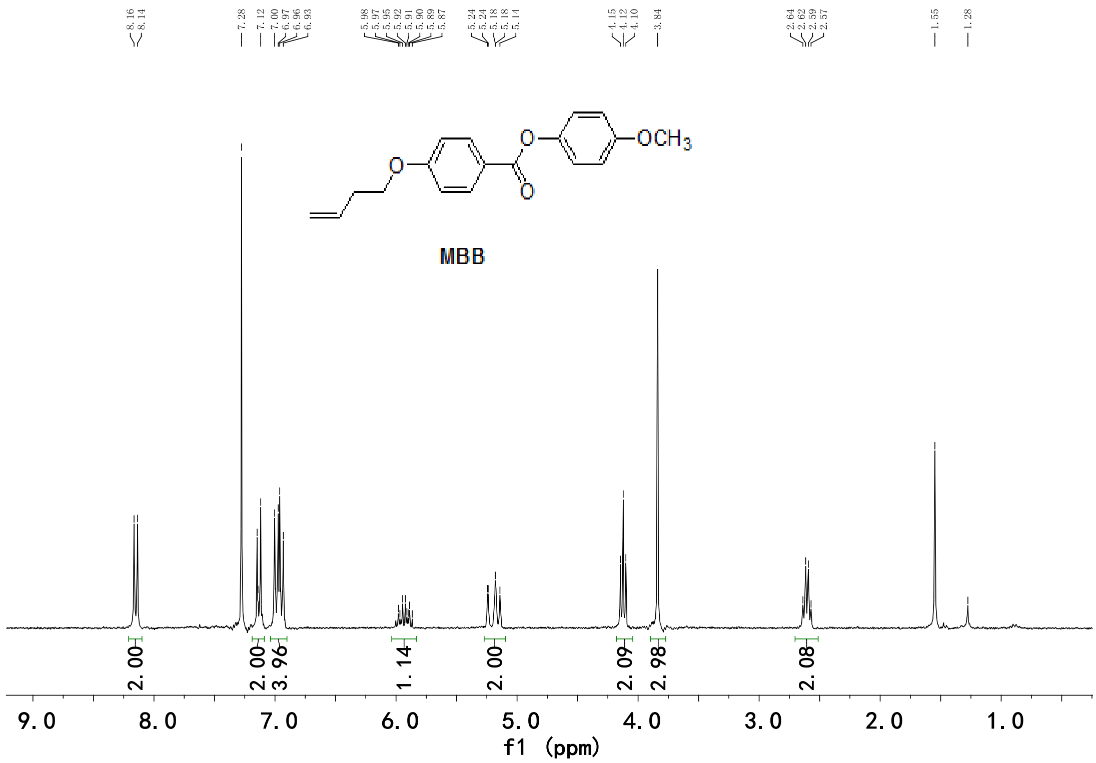

Supplementary Figure 13 | ^{1}H NMR spectrum of MBB.

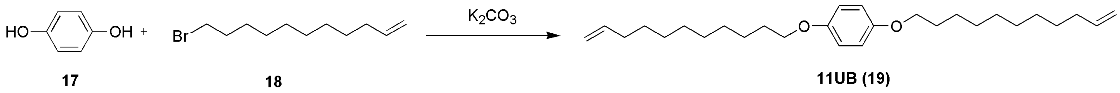

Supplementary Figure 14 | Synthetic route of crosslinker 11UB.

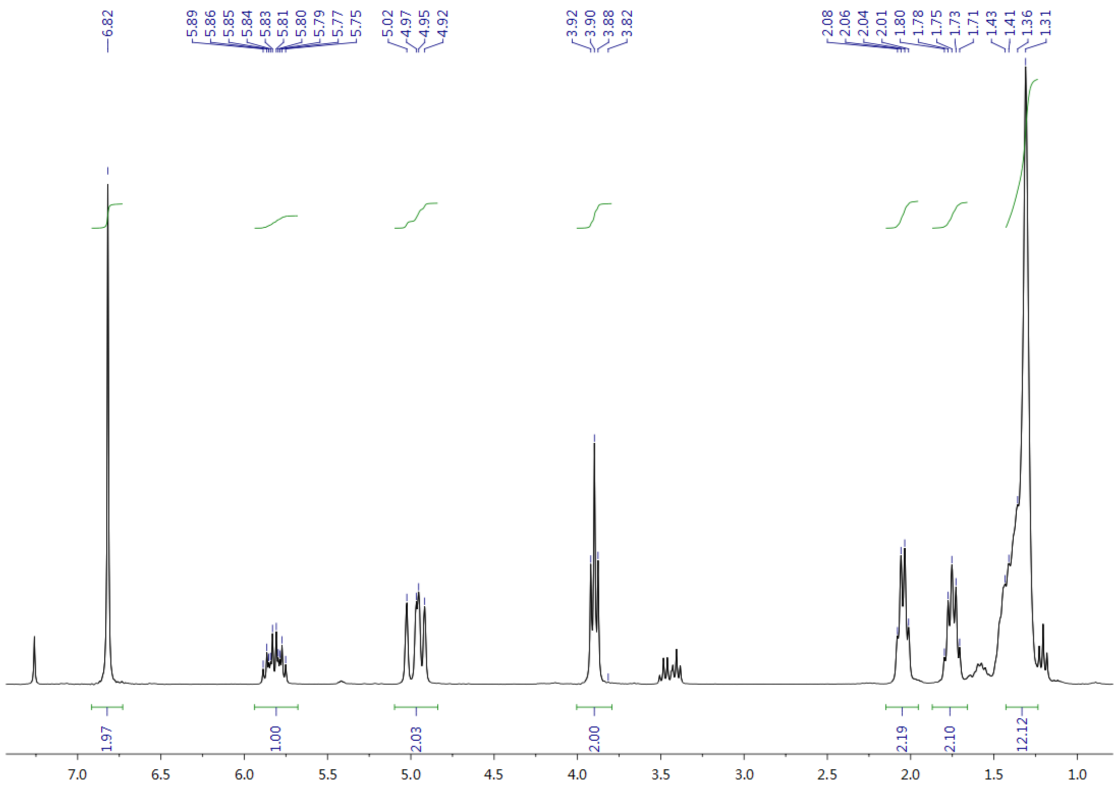

Supplementary Figure 15 | ^{1}H NMR spectrum of crosslinker 11UB.

Supplementary Methods

General Considerations. Polymethylhydrosiloxanes (HMS-993, M.W. 2200-2400) were purchased from Gelest Inc. Croconic acid was purchased from Alfa Aesar Inc. Diisopropyl azodicarboxylate (DIAD), Dicyclohexylcarbodiimide (DCC), triphenyl phosphate(TPP) 1,3-diisopropylcarbodiimide (DIC) and dimethylaminopyridine (DMAP) were purchased from Aladdin Inc. Dichloro(1,5-cyclooctadiene)platinum(II) was purchased from TCI (Shanghai,China). CH_2Cl_2 and THF were purified by a routine procedure and distilled from sodium benzophenone ketyl under nitrogen. All other chemicals were commercially analytical grade and used without further purification. All non-aqueous reactions were conducted in oven-dried glasswares, under a dry nitrogen atmosphere. Macherey-Nagel MN Kieselgel 60 (0.063-1.2 mm) was used on all flash chromatography. The Pt-catalyst solution was prepared by dissolving 0.025g of dichloro(1,5-cyclooctadiene) platinum(II) in 20 mL of CH_2Cl_2.

The ^{1}H NMR and ^{13}C NMR spectra of all monomers were obtained using a Bruker HW300 MHz spectrometer (AVANCE AV-300) to determine molecular structure. High-resolution mass spectra were obtained with Waters Micromass Q-TOF micro system mass spectrometer in positive ion mode. A TA Instruments Q100 instrument (New Castle, DE) was used to record differential scanning calorimetry (DSC) spectra under nitrogen purge at a heating rate of 10 °C/min from −40 to +120 °C, and to measure the various transition temperatures of the materials. A TU-1810 ultraviolet-visible spectrophotometer (UV/VIS spectrometer, Beijing Purkinje General Corp., China) was used to obtain the UV-Vis absorption spectra.

The UV/vis-responsive behavior experiments were performed by successively using a LP-20A UV lamp (5 mW·cm^{-2}, λ = 365 nm; LUYOR Corporation) and a CEL-HXF300 xenon lamp (Output power: 18.6 W, CEAULIGHT Corporation) to irradiate the sample. All NIR-repsonsive experiments were performed using a 808 nm semiconductor laser source (Output power: 8 W, Center wavelength: 808 ± 3 nm, Nanjing Latron Laser Company, China).

General procedure for preparation of a pre-crosslinked PMHS-MBB/YHD796 composite film. PMHS (24.0 mg, 0.400 mmol Si−H groups), MBB (104.0 mg, 0.349 mmol), 11UB (13.6 mg, 0.033 mmol) and YHD796 (0.7 mg, 0.5 wt %) were solved in 2 mL of toluene and high-power ultrasonicated for 5 min to ensure homogeneous dispersion. The mixture solution was cast into a polytetrafluoroethylene (PTFE) rectangular mold (2.0 cm long × 2.0 cm wide × 1.5 cm deep). After adding 80 μL of pre-prepared Pt-catalyst solution the PTFE mold was ultrasonicated for 5 min to remove the bubbles in the mixture solution and then heated in an oven at 60 °C for 4 h to accomplish the first cross-linking stage. After cooling to room temperature, the LCE sample was carefully removed from the PTFE mold with the help of hexanes, and then immediately cut into a strip (2.0 cm long × 0.5 cm wide, the thickness was ca. 0.1 mm), which was uniaxially stretched to ca. 140 ~ 160% of the original length, fixed by using tapes, and dried at room temperature.

General procedure for preparation of a pre-crosslinked

PMHS-AZO46-MBB/YHD796 composite film. The similar first-crosslinking process was performed. PMHS (24.0 mg, 0.400 mmol Si−H groups), MBB (76.0 mg, 0.254 mmol), 11UB (13.6 mg, 0.033 mmol), AZO46 (30.0 mg, 0.085 mmol) and YHD796 (0.7 mg, 0.5 wt %) were solved in 2 mL of toluene. The mixture was cast into a PTFE rectangular mold (2.0 cm long × 2.0 cm wide × 1.5 cm deep) after high-power ultrasonication for 5 min. After adding 80 μL of the pre-prepared Pt-catalyst solution into the above mixture, the PTFE mold was ultrasonicated for 5 min to remove the bubbles in the mixture solution and then heated in an oven at 60 °C for 4 h to accomplish the first cross-linking stage. After cooling to room temperature, the LCE sample was carefully removed from the PTFE mold with the help of hexanes, and then immediately cut into a strip (2.0 cm long × 0.2 cm wide, the thickness was ca. 0.1 mm), which was uniaxially stretched to ca. 140 ~ 160% of the original length, fixed by using tapes, and dried at room temperature.

General procedure for preparation of the bilayer LCE ribbons. The hydrosilylation reaction was performed to stick two partial-crosslinked layers. The two partial-crosslinked stripes were overlapped with a crossed angle θ of 45° or -45°. The top layer was extended YHD796/PMHS-MBB-AZO composite film and the bottom layer was extended YHD796/PMHS-MBB composite film. The fixed dual-layer LCE films were heated at 60 °C in an oven for 72 h to complete the second cross-linking stage. Then, the dual-layer film was cut into strips (2.0 cm long × 0.2 cm wide, the thickness is ca. 0.1−0.2 mm).

Synthesis of monomer YHD796 (Supplementary Figure 3). Step 1: Methyl isonipecotate (0.56 g, 3.90 mmol) and thiophene-2-thiol (0.33 g, 2.79 mmol) were dissolved in 4 mL of toluene. The mixture solution was heated to reflux for 3 h under nitrogen atmosphere. After cooling to room temperature, the mixture was diluted with 5 mL of ethyl acetate. After evaporation of the solvent, the residual light-yellow solid was purified by column chromatography on silica gel using petroleum ether/ethyl acetate (10/1) as eluent to give the desired product 3 (0.40 g, yield: 63 %) as a white solid. ^{1}H NMR (300 MHz, $CDCl_3$): δ 6.77 (m, 1H), 6.60 (t, J = 5.0 Hz, 1H), 6.13 (t, J = 5.0 Hz, 1H), 3.73 (s, 3H), 3.52 (m, 2H), 2.84 (m, 2H), 2.44 (m, 1H), 2.02 (m, 2H), 1.93–1.82 (m, 2H). Step 2: Compound 3 (0.2 g, 0.89 mmol) were added into 5 mL of 0.5 N sodium hydroxide solution. The reaction mixture was heated to reflux for 3 h. After cooled to room temperature, 2 mL of aqueous acetic acid (conc. 10%) was added into the mixture to adjust pH to ~4. The precipitate product was collected by filtration and dried under vacuum, to give intermediate 4 as a white solid (0.15 g, yield 80.4%). ^{1}H NMR (300 MHz, $CDCl_3$): δ 6.78 (dd, J = 6.0, 3.0 Hz, 1H), 6.61 (d, J = 6.0 Hz, 1H), 6.14 (d, J = 3.0 Hz, 1H), 3.52 (dd, J = 9.0, 6.0 Hz, 2H), 2.94–2.76 (m, 2H), 2.48 (m, 1H), 1.96 (m, 4H). Step 3: Compound 4 (0.15 g, 0.72 mmol), n-decyl alcohol (0.14 g, 0.87 mmol), DCC (0.18 g, 0.85 mmol) and DMAP (0.005 g, 0.04 mmol) were dissolved into 5 mL of dry CH_2Cl_2. The solution was stirred at room temperature for 24 h under nitrogen atmosphere. After filtering off the precipitate, the solution was concentrated by rotary evaporation. The crude oil was purified by column chromatography on silica gel using petroleum ether/ethyl acetate (15/1) as

eluent to give the product 6 (0.14 g, yield 55.5%) as a colorless oil. ^{1}H NMR (300 MHz, $CDCl_3$): δ 6.77 (dd, J = 6.0, 3.0 Hz, 1H), 6.60 (t, J = 6.0 Hz, 1H), 6.13 (m, 1H), 4.16–4.03 (t, J = 8.3 Hz, 2H), 3.51 (m, 2H), 2.84 (m, 3.1 Hz, 2H), 2.42 (m, 1H), 2.07–1.87 (m, 4H), 1.40–1.22 (t, J = 11.7 Hz,16H), 0.89 (t, J = 6.6 Hz, 3H). Step 4: Compound 6 (0.14 g, 0.39 mmol), croconic acid 7 (0.03 g, 0.20 mmol) and 6 mL toluene/n-butanol (v/v, 1/1) were added into a 50 mL three-neck round-bottom flask. The reaction mixture was heated to reflux for 1 h under nitrogen atmosphere. After cooled to room temperature, the mixture was diluted with 3 mL dichloromethane. After evaporation of the solvent, the resulting crude black solid was purified by column chromatography on silica gel using CH_2Cl_2/methanol (50/1) as eluent to give the desired product YHD796 (0.13 g, yield 80%) as a black solid. ^{1}H NMR (300 MHz, $CDCl_3$): δ 8.67 (d, J = 45.0 Hz, 1H), 6.50 (s, 1H), 4.08 (m, 2H), 3.89 (s, 2H), 3.41 (s, 2H), 2.63 (s, 1H), 1.95 (t, J = 9.0 Hz, 4H), 1.68–1.51 (t, J = 9.0 Hz, 2H), 1.26 (d, J = 12.0 Hz, 14H), 0.85 (t, J = 6.0 Hz, 3H). All spectral data match those previously reported.[1]

Synthesis of monomer AZO46 (Supplementary Figure 8). Step 1: Concentrated HCl (1.05 mL, 12.2 mmol) and 4-butoxyaniline (1.00 g, 6.1 mmol) were added into 30 mL of PEG200/1,4-dioxane/water (v/v/v, 60/30/10) in an ice bath. After dropwise adding 2 mL of $NaNO_2$ aqueous solution (0.46 g in 10 mL water), the mixture was stirred at 5 ^{o}C for 1 h to form the diazonium salt (2). Phenol (1.71 g, 18.2 mmol) and NaOH (0.27 g, 6.7 mmol) were dissolved in 30 mL of PEG200/1,4-dioxane/water

(v/v/v, 60/30/10). The mixture was added into the diazonium salt solution and stirred for 15 min. Water (70 mL) was poured into the mixture and HCl was added to make the mixture slightly acidic (pH= 4~5). After filtration, the collected red powder was washed with water and dried in vacuum. The final purification was carried out by column chromatography using cyclohexane/ethyl acetate (90/10) to give the product 11 (1.52 g, Yield: 77.7%) as a red solid. ^{1}H NMR (300 MHz, $CDCl_3$): δ 7.92 (m, 3H), 7.05 (d, J= 6.0 Hz, 2H), 6.97 (d, J= 6.0 Hz, 2H), 5.55 (s,1H), 4.10 (t, J = 5.0 Hz, 2H), 1.88 (m, 2H), 1.60 (m, 2H), 1.06 (t, 3H, J = 6.0 Hz). Step 2: Phenol 11 (1.00 g, 2.16 mmol), 5-hexen-1-ol (0.43 g, 2.72 mmol) and TPP (0.85 g, 3.24 mmol) were added into 15mL of CH_2Cl_2. DIAD (0.56 g, 3.24 mmol) was added dropwise into the above solution in an ice bath and stirred at room temperature overnight. After evaporation, the collected solid was purified by column chromatography on silica gel using petroleum ether/ethyl acetate (4/1) as eluent. The product AZO46 was recrystallized twice from ethanol as yellow crystals; yield 0.90 g (69%). ^{1}H NMR (300 MHz, $CDCl_3$) δ: 7.87 (m, 4H), 6.99 (m, 4H), 5.88 (m, 1H), 5.06–4.97 (m, 2H), 4.05 (t, J = 6.0 Hz, 4H), 2.16 (m, 2H), 1.83 (m, 4H), 1.61-1.51 (m, 4H), 1.01 (t, J = 6.0 Hz, 3H). ^{13}C NMR (500 MHz, $CDCl_3$): δ 161.2, 161.1, 146.9, 138.4, 124.3, 114.8, 114.7, 68.1, 68.0, 33.4, 31.3, 28.7, 25.3, 19.2, 13.8.

Synthesis of monomer MBB (Supplementary Figure 12). But-3-en-1-ol (1.44 g, 20.0 mmol), 4-hydroxybenzoic acid ethyl ester (3.32 g, 20.0 mmol) and TPP (5.45 g, 20.0 mmol) were dissolved in 15 mL of THF. DEAD (3.48 mL, 20.0 mmol) was

added dropwise into the above solution in an ice bath over 10 minutes. The reaction mixture was stirred for 24 h at the room temperature under nitrogen atmosphere. After evaporation of the solvents, the crude product was purified by flash column chromatography on silica gel using ethyl acetate/petroleum ether (1/6) as eluent to provide compound 15 (3.85 g, yield: 87.5%) as a colorless oil. Compound 15 (3.85 g, 14.48 mmol) and sodium hydroxide (1.40 g, 35.95 mmol) were added into 50 mL of ethanol/water (v/v, 1/1). The mixture solution was heated to reflux for 12 h. After cooling to room temperature, 1 M hydrochloric acid solution was added into solution to adjust pH to ~ 2. . The white precipitate was collected by filtration and washed with water. The crude product was recrystallized from ethanol to give compound 16 as a white solid (3.25 g, yield: 96.8%). Compound 16 (13.00 g, 67.70 mmol), 4-methoxyphenol (9.23 g, 74.47 mmol), DMAP (4.13 g, 33.85 mmol) and DIC (10.23 g, 81.24 mmol) were dissolved in 140 mL of dry dichloromethane. The mixture solution was stirred at room temperature for 18 h. After evaporation the solvents, the resulting white solid was purified by flash column chromatography on silica gel using dichloromethane as the eluent. The crude product was recrystallized from ethanol to give the desired product MBB as white crystals (16.20 g, yield: 80.3%). ^{1}H NMR (300 MHz, $CDCl_3$): δ 8.15 (d, J = 6.0 Hz, 2H), 7.19–7.09 (m, 2H), 6.97 (m, 4H), 5.92 (m, 1H), 5.27–5.10 (m, 2H), 4.12 (t, J = 9.0 Hz, 2H), 3.84 (s, 3H), 2.60 (q, J = 6.0 Hz, 2H). All spectral data match those previously reported.[2]

Synthesis of crosslinker 11UB (Supplementary Figure 14). Hydroquinone (2.20 g, 20 mmol) and potassium carbonate (9.68 g, 70 mmol) were dissolved in 100 mL of ethanol. After dropwise adding 11-chloro-1-undecene (9.06 g, 48 mmol) into the above mixture over 30 minutes, the mixture solution was heated to reflux for 18 h. The reaction mixture was poured into 400 mL of iced water and extracted three times with CH_2Cl_2 (800 mL in total). The organic layer was washed twice with saturated sodium carbonate aqueous solution (200 mL in total) and once with water (200 mL), followed by drying over anhydrous $MgSO_4$. After evaporation of solvents, the residue was purified by flash column chromatography on silica gel using petroleum ether/ethyl acetate (10:1) as the eluent to give the desired product 11UB (12.04 g, yield 65%). ^{1}H NMR (300 MHz, $CDCl_3$): δ 6.80 (s, 2H), 5.80 (m, 2H), 4.98 -4.92 (m, 4H), 3.88 (t, J = 6.0 Hz, 4H), 2.01 (m, 4H), 1.73 (m, 4H), 1.18 (m,24H). All spectral data match those previously reported.[3]

Supplementary References

1. Guo, L. X. *et al.* A calamitic mesogenic near-infrared absorbing croconaine dye/liquid crystalline elastomer composite. *Chem. Sci.* **7**, 4400–4406 (2016).

2. Arehart S. V. & Pugh C. Induction of smectic layering in nematic liquid crystals using immiscible components. 1. Laterally attached side-chain liquid crystalline poly(norbornene)s and their low molar mass analogs with hydrocarbon/fluorocarbon substituents. *J. Am. Chem. Soc.* **119**, 3027–3037 (1997).

3. Agrawal, A. *et al.* Dynamic self-stiffening in liquid crystal elastomers. *Nat. Commun.* **4**, 1739 (2013).
